# Linking antibiotic resistance genes in the vaginal microbiota to health-related behaviors and antibiotic awareness in reproductive-age women: a cross-sectional study

**DOI:** 10.3389/fcimb.2025.1640992

**Published:** 2025-09-18

**Authors:** Paola Castellano, Camilla Ceccarani, Marielle Ezekielle Djusse, Michela Mazzetti, Sara Morselli, Tania Camboni, Silvia Conti, Federica Prinelli, Marco Severgnini, Claudio Foschi, Margherita Dall’Asta, Clarissa Consolandi, Antonella Marangoni

**Affiliations:** ^1^ Department of Medical and Surgical Sciences (DIMEC), Alma Mater Studiorum - University of Bologna, Bologna, Italy; ^2^ Institute of Biomedical Technologies, National Research Council, Segrate, Italy; ^3^ National Biodiversity Future Center S.c.a.r.l., Palermo, Italy; ^4^ Section of Microbiology, Department of Medical and Surgical Sciences (DIMEC), Alma Mater Studiorum - University of Bologna, Bologna, Italy; ^5^ International PhD College, Collegio Superiore of Alma Mater Studiorum, University of Bologna, Bologna, Italy; ^6^ Department of Medical Sciences, University of Ferrara, Ferrara, Italy; ^7^ Microbiology Unit, IRCCS Azienda Ospedaliero-Universitaria di Bologna, Bologna, Italy; ^8^ Department of Animal Science, Food and Nutrition (DIANA), Università Cattolica Del Sacro Cuore, Piacenza, Italy

**Keywords:** vaginal microbiota, antimicrobial resistance, ARG, resistome, women’s health

## Abstract

**Introduction:**

The vaginal microbiota (VMB), predominantly shaped by *Lactobacillus* species, plays a crucial role in maintaining vaginal health and preventing infections. However, its delicate balance is increasingly challenged by the widespread use of antibiotics and the consequent rise in antibiotic resistance genes (ARGs). While the VMB has been recognized as a potential reservoir for ARGs, limited research has explored how microbial composition, antibiotic exposure, and individual behavioral factors converge to shape the vaginal resistome.

**Materials and methods:**

In this cross-sectional study, vaginal swabs were collected from 105 reproductive-age Caucasian women. The VMB composition was characterized and classified into Community State Types (CSTs), by sequencing the hypervariable V3-V4 regions of the bacterial 16S rRNA gene. In order to highlight common patterns of abundance among taxa, a co-abundance groups (CAGs) analysis was performed. We assessed the distribution of 14 ARGs conferring resistance to macrolides, tetracyclines, beta-lactams, and quinolones along with two associated transposons by means of PCR. An overall composite ARGs score was also calculated. Participants completed detailed questionnaires assessing demographics and behavioral factors, with a particular focus on both health- and antibiotic-related behaviors. Statistical analyses examined associations between ARG prevalence, vaginal microbiome composition and relevant exposures.

**Results:**

CSTs I and III were the most prevalent, with the most frequently detected ARGs being *erm(F)*, *tet(M)*, *erm(B)*, *erm(A*), and *tet(W)*, each present in over 65% of participants. ARG presence was positively correlated with a higher vaginal microbiome alpha-diversity. Moreover, BV-associated bacterial taxa showed strong associations with ARGs, while *Lactobacillus* species generally exhibited negative correlations. Smoking, a higher body mass index (BMI), presence of *Candida* spp. and a history of antibiotic use were significantly associated with increased ARG prevalence, whereas oral contraceptive use and higher diet quality scores were negatively associated. Correlating together the relative abundances of the microbial CAGs and the presence/absence of specific ARGs, we found a positive association between several genes related to macrolide and tetracycline resistance and the *Gardnerella*-*Prevotella* CAG. Additionally, the *Gardnerella*-*Prevotella*, and the *Streptococcus* CAGs were positively correlated to the total ARG score, whereas the *L. crispatus/jenesenii/gasseri* CAG was negatively correlated.

**Conclusions:**

These findings underscore the role of the VMB as a dynamic reservoir of ARGs and highlight the influence of individual lifestyle and antibiotic-related behaviors on ARG dissemination in the vaginal niche. This supports the need for integrated public health strategies that combine antibiotic stewardship with targeted lifestyle and behavioral interventions, as well as the development of individualized therapeutic approaches.

## 1 Introduction

The vaginal microbiota (VMB) is typically characterized by a low bacterial diversity, in stark contrast to other mucosal sites such as the gut, where microbial diversity is high. In healthy, reproductive-aged women, the VMB comprises a variety of aerobic and anaerobic bacterial genera and species, with the genus *Lactobacillus* generally dominating. A *Lactobacillus*-dominated VMB supports vaginal health by preventing the colonization and overgrowth of pathogenic microorganisms, maintaining homeostasis and eubiosis, and has been associated with a lower risk of infections (Wang et al., 2024; Greenbaum et al., 2019; Ceccarani et al., 2019).

Next-generation sequencing of the hypervariable regions of the 16S rRNA gene has enabled detailed analyses of the composition of the VMB, leading to its classification into five main Community State Types (CSTs). Four of these are dominated by individual *Lactobacillus* species (CST I - *L. crispatus*, CST II - *L. gasseri*, CST III - *L. iners*, CST V - *L. jensenii*), while CST IV is characterized by a heterogeneous assemblage of strict and facultative anaerobes, including *Gardnerella*, *Atopobium*, *Mobiluncus*, and *Prevotella* (Borgogna et al., 2020; France et al., 2022).

Over a woman’s lifespan, the VMB, which plays a pivotal role in women’s health, can undergo significant changes in response to both local and systemic factors, potentially leading to dysbiosis. In literature, a long but non-exhaustive list of risk factors associated with vaginal dysbiosis has been reported. These include age, body mass index (BMI), hormonal status, diet, ethnicity, smoking, lifestyle, antibiotic use, and the presence of urogenital infections. Although essential for treating bacterial infections, antibiotics can inadvertently disrupt the delicate vaginal microbial ecosystem, resulting in negative health consequences (Abou Chacra et al., 2022; Ahrens et al., 2020; Morsli et al., 2024; Das et al., 2023).

The global increase in antibiotic use, often beyond clinical indications, has sparked concern regarding its long-term impact on human health. In particular, the overuse and misuse of antibiotics has contributed to the emergence of antibiotic-resistant bacteria and the spread of antibiotic resistance (AR), posing a significant threat to public health. Tetracyclines are the most common antibiotics in agricultural soil and food production, due to their widespread usage and strong persistence (Wang et al., 2024). Moreover, tetracyclines together with other antibiotic classes, such as macrolides and quinolones, which disrupt DNA and protein synthesis, have displayed *in vitro* activity against different pathogens, thus becoming the recommended drugs for clinical treatment in different settings (Georgakopoulou et al., 2024). Selective pressure exerted by environmental antibiotics facilitates the spread of AR through horizontal gene transfer, predominantly via mobile genetic elements such as plasmids and transposons (Sirichoat et al., 2020; Flórez et al., 2016).

In particular, the link between antibiotic use and the emergence of resistance in the VMB is of special concern, since the VMB has indeed been identified as a significant reservoir of AR determinants (Jeters et al., 2009; Severgnini et al., 2021). Moreover, the increasing prevalence of antibiotic resistance genes (ARGs) in commensal bacteria is problematic, as these organisms can serve as reservoirs, transferring resistance determinants to pathogenic species (Melkumyan et al., 2015).

Individuality and social aspects play a role in antibiotic adherence behavior. About the first, both the fear of illness and the lack of knowledge about how antibiotics function (Nortey et al., 2023) lead to the misuse of antibiotics. In terms of sociality, the family also represents an environment capable of affecting the health behaviors of individuals and situations of unhealthy practices (e.g., drug consumption) (Bagley et al., 2022).

A recent study highlighted the impact of family experiences and individual attitudes on antibiotic-related behaviors (Castellano et al., 2024), underscoring the need to explore how these psychosocial elements influence the presence of ARGs in the vaginal environment.

In this context, it’s important to investigate the distribution of resistance genes in the VMB, the relationship between antibiotic consumption and the presence of ARGs in the vaginal milieu, and how individual practices, awareness, and knowledge regarding antibiotic use may influence the VMB and the prevalence of ARGs.

In a previous paper (Severgnini et al., 2021), we assessed the presence of only 4 resistance markers (i.e., *erm(B)* and *erm(F)* conferring resistance to macrolides; *tet(W)* and *tet(M)* to tetracyclines) in the vaginal environment of women at different gestational ages.

With the aim of expanding and further exploring the dynamics of the vaginal environment, in this cross-sectional study, we assessed the distribution of a wider panel of ARGs (n=14) conferring resistance to macrolides, tetracyclines, beta-lactams and quinolones, along with two associated transposons, across vaginal CSTs in non-pregnant reproductive-age women. In particular, the presence of the following determinants of resistance was evaluated: *erm(A)*, *erm(B)*, *erm(F)*, *tet(M)*, *tet(M)-Tn916*, *tet(O)*, *tet(W)*, *tet(Q)*, *tet(Q)-rteA*, *blaOXA-2*, *blaTEM*, *blaZ*, *blaSHV*, *blaCTX-M*, *qnrA* and *qepA*. As an innovative aspect, we also examined potential factors (exposures) associated with the presence of these genes and the overall composite ARGs score, with a particular focus on individual knowledge, attitudes, and habits regarding antibiotic use, employing a multivariate approach. The detection of ARGs was subsequently correlated with the bacterial composition of the vaginal microbiome.

## 2 Materials and methods

### 2.1 Study setting and population

From November 2023 to April 2024, 123 Caucasian women of reproductive age were enrolled in the study. The participants were volunteer students attending degree courses at the University of Bologna, Italy. At enrolment, the exclusion criteria were: (i) antibiotic use in the month prior to sampling; (ii) use of vaginal douches or topical agents in the last two weeks; (iii) age < 18 years; (iv) pregnancy; (v) menstruating at the time of sampling; (vi) HIV infection; (vii) presence of chronic conditions (e.g., diabetes, autoimmune diseases, malignancies); (viii) drug addiction or heavy smoking (>15 cigarettes/day). Moreover, women with urogenital infections due to sexually transmitted pathogens (i.e., *Chlamydia trachomatis*, *Neisseria gonorrhoeae, Trichomonas vaginalis, Mycoplasma genitalium*) were further excluded when diagnostic test results were available.

Several demographic (e.g., BMI, educational level, marital status), health-related (e.g., presence of *Candida* spp., smoking habits) and antibiotic-related behavioral (e.g., individual practice and awareness about antibiotics) data were collected (see specific paragraphs on exposures).

After obtaining written informed consent from all participants, they underwent two self-collected vaginal samplings at the same time point. The first swab (E-swab, Copan, Brescia, Italy) was used for diagnostic tests to exclude the presence of sexually-transmitted infections (STIs) by NAAT (Nucleic Acid Amplification Test, Alinity m STI Assay, Abbott Molecular Inc, Des Plaines, IL, USA). The second was collected with a sterile cotton bud, re-suspended in 1 mL of sterile saline, and stored at -80 °C until use.

The study was conducted in accordance with the Declaration of Helsinki and approved by the Institutional Review Board (IRB) of the University of Bologna, protocol number 0122421.

### 2.2 Amplification of the various ARGs

Nucleic acids were extracted from vaginal swabs by means of the DNeasy Blood & Tissue Kit (QIAGEN GmbH, Hilden, Germany) according to the manufacturer’s instructions. From the DNA eluate, each sample was screened for the presence of selected resistance genes and specific transposons that confer resistance to different classes of antibiotics by means of end-point PCR assays. The following genes were investigated: *erm(A)*, *erm(B)* and *erm(F)* conferring resistance to macrolides; *tet(M)*, *tet(O)*, *tet(W)*, and *tet(Q)*, conferring resistance to tetracyclines; *blaOXA-2*, *blaTEM*, *blaZ*, *blaSHV* and *blaCTX-M* associated with β-lactam resistance; and *qnrA* and *qepA* linked *to* quinolone resistance. Additionally, the presence of two transposons associated with AR, namely *tet(M)-Tn916 and tet(Q)-rteA*, was also assessed. Detailed information on primers used and PCR conditions are displayed in the **Supplementary Materials** (**Supplementary Table S1**). These results were dichotomized as 0=absence of antibiotic resistance and 1=presence of antibiotic resistance.

An overall composite score for the ARGs was developed by summing the presence/absence of all the genes detected in at least one sample. The total possible score ranged from 0 to 10, with higher scores indicating greater antibiotic resistance. The score was also categorized into tertiles based on its distribution: low (≤3), medium (4-5), and high (≥6).

### 2.3 Vaginal microbiome molecular profiling

The hypervariable V3-V4 regions of the bacterial 16S rRNA gene were amplified from genomic DNA extracted from vaginal swabs. The PCR conditions and primer sequences were obtained from the Illumina 16S Sample Preparation Guide (https://support.illumina.com/documents/documentation/chemistry_documentation/16s/16s-metagenomic-library-prep-guide-15044223-b.pdf) (Illumina, San Diego, CA, USA), with the primers originally described in Klindworth et al., 2013. Final indexed libraries were prepared by equimolar (4 nmol/L) pooling, denaturation, and dilution to 6 pmol/L before loading on a MiSeq flow cell (Illumina) for a 2 × 300 bp paired-end run. 16S rRNA sequences were processed according to the same methods as described in Severgnini et al., 2021, using the latest release of the SILVA database (v. 138, Quast et al., 2013) for classification.

Depth of sequencing was set to the lowest sequenced sample (n=6,627 reads), in order to compensate for the sequencing unevenness of the samples and to provide a consistent minimum amount for the downstream analysis, carried out through the QIIME pipeline (version 1.9.0, Caporaso et al., 2010). Alpha-diversity was estimated according to Chao1, Shannon Index, Observed Species, and the Faith’s phylogenetic tree diversity metrics (“PD whole tree”), whereas beta-diversity was evaluated using unweighted and weighted UniFrac distances (Lozupone et al., 2011). Since all alpha-diversity indices performed similarly, we focused on the PD whole tree for the univariate and multivariate analyses (see below).

*Lactobacillus* species-level characterization was performed as in Severgnini et al., 2022 by BLAST-aligning all reads belonging to the Lactobacillaceae family to a custom reference database made up of all available reference sequences in the NIH-NCBI database (https://ftp.ncbi.nlm.nih.gov/genomes/GENOME_REPORTS/prokaryotes.txt) of 17 species commonly found in the vaginal environment, including a total of 3,392 genomes. In case of multiple matches with the same confidence, the taxonomy was reset to “Unclassified *Lactobacillus*” at the genus level.

Community-state types (CST) of the vaginal microbial communities were determined from the taxonomic profiles using VALENCIA, a nearest centroid-based tool that classifies samples into 5 major CST according to the similarity to a set of about 13,000 reference microbial profiles (France et al, 2020).

In order to highlight common patterns of abundance among taxa, a co-abundance groups (CAGs) analysis was performed as previously described (Claesson et al, 2012). Briefly, only the bacterial taxa present at >0.5% of abundance in at least 10% of the samples (n≥11) were selected, in order to exclude minor and transient contributors of the gut microbiota. This resulted in a subset of 21 taxa on the whole dataset. The co-abundance between each pair of taxa was evaluated by calculating Spearman’s correlation coefficient and displayed in a heatmap, hierarchically clustered using Pearson’s correlation metric and average linkage. Only associations having a Benjamini-Hochberg adjusted p<0.05 were used to build the hierarchical clustering. Permutational multivariate analysis of variance (P-MANOVA) was used to assess that the computed CAGs were significantly different from each other, using p<0.05 as a threshold.

### 2.4 Exposures

For the present analysis, exposures were selected based on theoretical knowledge because they are crucial for contextualizing the results, as they could potentially influence both antibiotic use and health outcomes (Morsli et al., 2024). These factors included the following demographic, health- and antibiotic-related behavioral data of the participants: age (continuous), educational level (categorized as high school *vs* university degree or higher), marital status (married/cohabiting *vs* single/separated), body mass index (BMI, calculated as weight in kg divided by height in cm squared), contraceptive use, smoking habits (non-smokers *vs* smokers), presence/absence of *Candida* spp., adherence to a healthy diet such as the Mediterranean diet (measured calculating the MEDILITE score, Sofi et al., 2017). In addition, we investigated individual practice and awareness about antibiotics and AR, and participants’ family of origin approach and behavior toward antibiotics, through *ad hoc* questions, to identify possible misuse of these drugs. An adapted version of the “Antibiotic Resistance: Multi-Country Public Awareness Survey,” developed by the World Health Organization (WHO, 2015), was employed. This revised survey consisted of 25 items organized into several sections. The first section contained two questions regarding the amount of antibiotics taken in the last year and month, along with four questions focused on best practices for antibiotic use where participants’ individual scores were derived from the sum of their answers. The second section included nine statements aimed at assessing participants’ understanding of antibiotic resistance, with responses recorded as True or False. Individual scores were calculated by summing the responses. The final section comprised ten statements evaluating participants’ beliefs and attitudes towards antibiotic use and resistance, utilizing a 5-point Likert scale (1 = strongly disagree to 5 = strongly agree). The total scores from these items were used as individual scores to gauge “awareness,” which refers to the level of self-awareness and understanding regarding strategies to combat AR. Eight *ad hoc* questions were included to investigate participants’ family background and attitudes towards antibiotics, with the aim to identify family models which used antibiotics appropriately (e.g., avoiding self-prescribing and adhering to prescribed dosages). In order to better explore the variable related to family antibiotic improper use, we categorized the total score in tertiles based on the distribution as low (≤10), medium (11–17) and high (≥18).

### 2.5 Statistical analysis

Sample characteristics were summarized using mean and standard deviation (SD) for continuous variables and counts and percentages for categorical variables.

Statistical evaluation of alpha-diversity indices for the microbiota profiles was performed by non-parametric Monte Carlo-based tests. Beta-diversity differences were assessed by a permutation test with pseudo F-ratios using the “adonis” function from R package “vegan” (version 2.0-10, https://cran.r-project.org/package=vegan). Relative abundances were analyzed first setting all “0” to a value of 10^–6^ in order to compensate for missing data due to the normalized ASV table (where the “0” can be due to insufficient depth of coverage for assessing the ASV presence) and, then, performing a centered log-ratio (CLR) transformation in order to deal with the compositional nature of the data; finally, a Kruskal-Wallis test, followed by a Dunn *post hoc* evaluation was performed. Correlation between presence of specific ARGs and bacterial taxa abundances was performed by point-biserial correlation, whereas correlation between the total ARG score and bacterial taxa abundances was based on the Spearman’s correlation coefficient (with a p-value ≤ 0.05 considered as significant).

Two regression models were performed to identify factors associated with antibiotic-resistance genes. Binary logistic regression models were constructed to calculate odds ratios (ORs) and 95% confidence intervals (CIs) to estimate the association between exposures of interest and the presence of each of the antibiotic resistance genes.

Linear regression models were used to calculate the exponentiated β-coefficients with 95% CIs to examine the associations between exposures and the continuous antibiotic-resistance gene score.

We first performed univariate analyses including all the following variables in the model separately: age, educational level, marital status, smoking habits, BMI, presence of *Candida*, family history of inappropriate antibiotic use, antibiotics taken in the past year, past compliance with antibiotic use, awareness of antibiotic resistance, adherence to MEDILITE, and biodiversity index (PD whole tree). Multivariate models were performed, including factors associated with a p-value ≤ 0.10 in univariate analyses. All analyses were performed with STATA (version 15.0, StataCorp LP, College Station, Texas, USA) and Matlab (v 2008b, Natick, MA, USA) and the functions from the Fathom Toolbox (Jones, 2017). Two-sided p-values of 0.05 and 0.10 were considered statistically significant or borderline significant, respectively.

### 2.6 Data availability

Raw sequencing data for this project are available in NCBI Short-Read Archive (SRA) under accession number PRJNA1188525 (https://www.ncbi.nlm.nih.gov/bioproject/PRJNA1188525).

## 3 Results

### 3.1 Study population and exposures

A total of 123 participants were initially recruited. After excluding individuals with missing vaginal microbiota data (n = 7) and/or incomplete antibiotic behavior questionnaires (n = 14), 105 women were included in the final analysis. The mean age of participants was 21.6 ± 2.6 years (range: 19–30), and the mean BMI was 21.5 ± 2.8 kg/m² (range: 16.2–30.1).

Vaginal samples were categorized into the five main CSTs according to their microbial composition: CST I (41.9%, n = 44), CST II (5.7%, n = 6), CST III (30.5%, n = 32), CST IV (17.1%, n = 18), and CST V (4.8%, n = 5).

Many participants reported some level of inappropriate antibiotic use within their family of origin, with 32.4% indicating a high tendency toward misuse. Regarding individual antibiotic consumption, 42.9% of participants reported not having taken antibiotics in the previous year.

Overall, participants demonstrated high awareness of antibiotic resistance (mean score: 42 ± 4), along with strong adherence to recommended antibiotic use practices (mean score: 9 ± 2). Baseline characteristics of the sample, including behavioral factors and antibiotic-related variables, are presented in **Table 1**.

**Table 1:** Participants’ baseline characteristics for sociodemographic, behavioral, and antibiotic-related variables (n=105).

		N	%	Mean	SD
Characteristics					
Age				21.6	2.6
Education	High school	75	71.4		
	University degree or higher	30	28.6		
Civil status	Married/cohabitant	13	12.4		
	Single	92	87.6		
Smoking	Non-smokers	74	70.5		
	Smokers	31	29.5		
Body mass index				21.5	2.8
Oral contraceptive use		25	23.8		
Antibiotic-related behaviors					
Family antibiotic improper use	Low (≤10)	39	37.1		
	Medium (11–17)	32	30.5		
	High (≥18)	34	32.4		
Antibiotics taken in the past year	None	45	42.9		
	One	34	32.4		
	Two	26	24.8		
Past compliance of antibiotic usage (mean, SD)				9	2
Awareness about antibiotics resistance (mean, SD)				42	4
Total score of antibiotic-resistance gene, continuous (mean, SD)				4.48	0.20
Total score of antibiotic-resistance gene, categorical	Low (≤ 3)	33	31.4		
	Medium (4–5)	35	33.3		
	High (≥6)	37	35.2		
Vaginal microbiota data					
*Candida* spp.		7	6.7		
Community state types	I-II-V	55	52.4		
	III	32	30.5		
	IV	18	17.1		
PD whole tree (mean, SD)				5.0	1.8

### 3.2 Detection of ARGs

Regarding ARGs distribution, *erm(F)* was the most detected one, with 72.2% of positivity (80 cases), followed by: *tet(M)* (74.3%, 78 cases), *erm(B)* (68.6%,72 cases), *erm(A)* (66.7%, 70 cases), *tet(W)* (65.7%, 69 cases), *blaZ* (36.2%, 38 cases), *tet(Q)* (35.2%, 37 cases), and *blaTEM* (16.2%, 17 cases). Furthermore, the less frequently detected genes were *blaOXA-2* and *tet(O)*, both with 2.9% of positivity (3 cases), *qnrA* with 1.9% (2 cases) and *qepA* with only 1% (1 case). No samples tested positive for *blaSHV* or *blaCTX-M*.

Positivity for specific ARGs was frequently associated with the presence of the corresponding mobile genetic elements. Specifically, *tet(Q)-rteA* was identified in 100% of the *tet(Q)*-positive samples (37/37), and *tet(M)-Tn916* was detected in 96.2% of the *tet(M)*-positive samples (75/78).

### 3.3 Factors associated with ARGs

We initially performed univariate analyses to explore potential associations between selected factors and the presence of ARGs (data not shown). Variables with a p-value ≤ 0.10 were subsequently included in multivariate models to assess their independent association with the outcomes of interest.

Several associations remained statistically significant in the multivariate analyses (**Table 2**). In detail, we found that the microbial alpha-diversity, estimated by the Faith’s phylogenetic distance metric (PD whole tree), was positively associated with *tet(M)*, *tet(O)*, *tet(Q)*, *erm(A)*, and *blaZ*, with odds ratios ranging from 1.57 to 2.22. Faith’s PD was also strongly associated with the total ARG score (Exp(β-coefficient) 1.82, 95%CI 1.53-2.17), whereas borderline significance was observed with *blaTEM* (p-value ≤ 0.10).

**Table 2:** Associations between exposures and the presence of ARGs.

Multivariate analysis	Macrolides						Tetracyclines									Beta-lactams									Total score of resistance genes		
	*erm(B)*			*erm(A)*			*tet(M)*			*tet(O)*			*tet(Q)*			*blaTEM*			*blaOXA-2*			*blaZ*					
	OR	95%CI		OR	95%CI		OR	95%CI		OR	95%CI		OR	95%CI		OR	95%CI		OR	95%CI		OR	95%CI		Exp(β)	95%CI	
Education (University degree or higher)				0.51	0.19	1.37																					
Contraception (yes *vs* no)							0.30**	**0.10**	**0.89**																		
Smoking (yes *vs* no)	3.47**	**1.08**	**11.18**													2.32	0.76	7.12							2.09**	**1.04**	**4.20**
Body mass index (one-unit increase)				1.25**	**1.01**	**1.55**				1.63*	**0.99**	**2.71**							1.28	0.95	1.72				1.13**	**1.01**	**1.26**
MEDILITE (one-unit increase)										0.09**	**0.01**	**0.99**													0.93	0.82	1.07
*Candida* spp. (yes *vs* no)													3.87	0.46	32.29							3.45	0.73	16.31	3.85**	**1.12**	**13.17**
Family Antibiotic Improper Use (one-unit increase)				1.08**	**1.01**	**1.15**				1.10	0.90	1.33							1.08**	**0.99**	**1.18**						
Antibiotics taken in the last year (one-unit increase)							2.00*	**0.95**	**4.19**										1.55	0.47	5.14						
Past compliance (one-unit increase)	0.69*	**0.46**	**1.02**				0.47**	**0.28**	**0.80**													0.75**	**0.59**	**0.97**	0.80*	**0.64**	**1.01**
Awareness about antibiotic resistance (one-unit increase)													0.86**	**0.76**	**0.98**										0.98	0.90	1.08
PD whole tree (one-unit increase)	1.51**	**1.09**	**2.09**	1.75**	**1.32**	**2.32**	2.22**	**1.51**	**3.28**	14.36**	**1.74**	**118.29**	1.69**	**1.25**	**2.27**	1.31*	**0.98**	**1.78**	1.79	0.82	3.90	1.57**	**1.18**	**2.09**	1.82**	**1.53**	**2.17**

Multivariate models were performed including factors associated with a p-value of ≤0.10 in the univariate analysis. Variables associated with antibiotic resistance genes are in bold, with a p-value of ≤0.05 (**) or ≤0.10 (*).

Contraceptive use reduced the odds of *tet(M)* by 70% compared to non-use. Smoking increased the odds of *erm(B)* by 3.47 times and the Exp(β-coefficient) of the total score increased by 2.09 times. A higher BMI was positively associated with *erm(A)* and a higher total ARG score, with a borderline positive association with *tet(O)*. Diet quality, as assessed by the MEDILITE score, was inversely associated with *tet(O)* detection, suggesting a potential protective effect of adherence to a Mediterranean diet. The presence of *Candida* spp. was significantly associated with a higher total ARG score (OR = 3.85).

Regarding antibiotic behavior, a higher degree of inappropriate antibiotic use in the family was associated with a slight increase in the odds of *erm(A)* and a borderline association with *blaOXA-2*. Antibiotic use in the past year was positively associated with *tet(M)*. Past compliance of antibiotic usage mainly reduced the odds of *tet(M)* and *blaZ*. Moreover, a borderline inverse association with *erm(B)* and the total score was also noticed (with odds ratios ranging from 0.47 to 0.80). Awareness about antibiotic resistance decreased the odds of *tet(Q)* by 14%.

### 3.4 Analysis of the vaginal microbiota associated with ARGs

The multivariate analysis indicated that the alpha-diversity of the vaginal microbiota (according to the PD whole tree metric) was positively associated with the presence of several ARGs. At the same time, the microbial profiles between samples positive or negative to several ARGs were found to be statistically different. In particular, we confirmed the different microbial composition of samples positive/negative for *erm(A)*, *erm(B)*, *tet(M)*, *tet(O)*, *tet(Q)*, and *blaZ* (unweighted UniFrac distance, p ≤ 0.008, adonis test of pseudo F-ratios). Moreover, also other ARGs (i.e.: *erm(F)* and *tet(W)*) had different microbial profiles (p=0.001 and p=0.002, respectively, unweighted UniFrac), whereas *blaTEM*, borderline associated to biodiversity, showed only a trend towards separation (p=0.191 unweighted UniFrac). Finally, microbial profiles for *blaOXA-2*, *qnrA* and *qepA* were not significantly different, due to the very low fraction of samples positives for these ARGs (n=3, n=2 and n=1, respectively) (**Supplementary Figure S1** in the **Supplementary Materials**).

**Figure 1:**
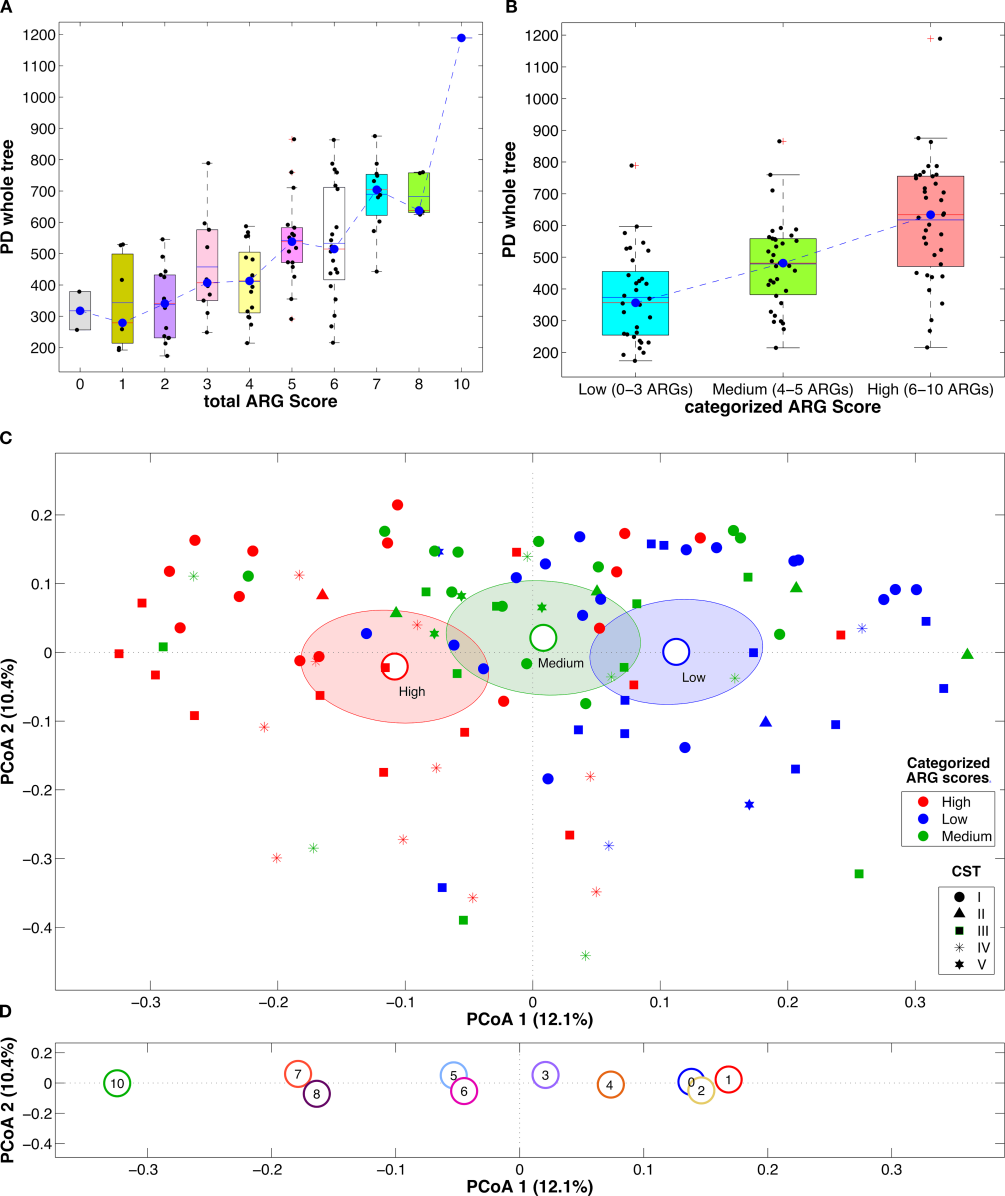
Boxplots of the alpha-diversity estimation according to Faith’s phylogenetic diversity metric with the samples grouped according to the total ARG score **(A)** or the total ARG score as categorized in tertiles **(B)**. Blue dashed lines represent the trend of the distribution medians; boxplots report the average (blue solid line) and median (red solid line) and individual samples as dots superimposed to the box-and-whiskers plots. Principal coordinate analysis (PCoA) plots based on the unweighted UniFrac distance among samples. Points represent single samples, colored, in each plot, according to the total ARG score as categorized in tertiles **(C)** or the total ARG score **(D)**; marker shapes for the single samples are represented according to the sample CST; centroids represent the average coordinate of the samples within the same category and ellipses are the SEM-based confidence intervals; for each plot, the first and second coordinate are represented.

Interestingly, significant changes in both alpha- and beta-diversity were found when analyzing the vaginal microbiota and the number of ARGs per sample. In fact, a clear trend towards an increase in alpha-diversity with increasing ARGs was evident, both considering the total ARG score and its categorization into tertiles. Moreover, the beta-diversity analysis showed a significant separation among the samples according to the ARG score tertiles (p<0.008 and p<0.024 for all pairwise comparisons, unweighted and weighted UniFrac distances, respectively) and also a pattern along principal coordinate 1 moving from the right to the left of the x-axis with increasing total ARG scores (**Figure 1**).

**Figure 2:**
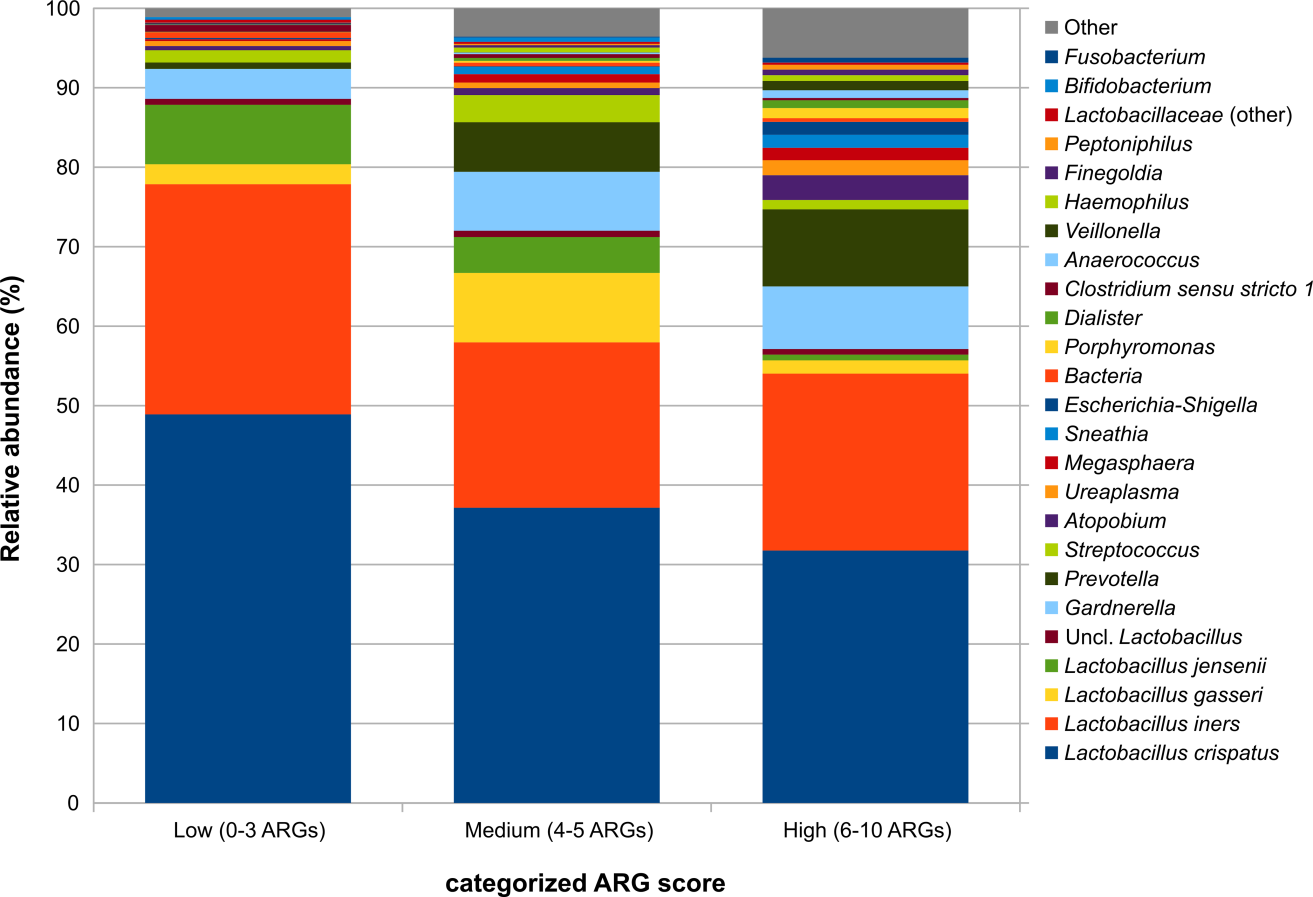
Barplots representing the average relative abundance for the main bacterial taxa (average rel. ab >0.5% in at least one experimental group) over the samples grouped by the categorized total ARG score. The score was categorized into tertiles based on its distribution: low (≤3 ARGs), medium (4–5 ARGs), and high (≥6 ARGs).

Considering the bacterial taxa involved in the vaginal microbiota, the abundance of some genera such as *Prevotella*, *Porphyromonas*, *Dialister*, *Finegoldia*, and *Peptoniphilus* were significantly (p<0.05, Kruskal-Wallis and Dunn’s *post hoc* pairwise tests) increased in both the “medium” and the “high” categorized ARG score tertiles as compared to “low”, whereas *Gardnerella*, *Streptococcus*, *Megasphaera*, *Haemophilus*, *Anaerococcus*, and *Fusobacterium* increased in “high” score samples only (**Supplementary Figure S2** in the **Supplementary Materials**). Species from the *Lactobacillus* genus did not show a significant decrease with increasing total ARG scores but, as a whole, they accounted for about 90% of the relative abundance in “low” samples, while shifting to about 70% in “medium” and to about 55% in “high” ones (**Figure 2**).

### 3.5 Correlation between bacterial taxa and ARGs

Co-abundance analysis highlighted the presence of 4 groups (CAGs) with a similar pattern of abundance of their members over all the samples (**Supplementary Figure S3** in the **Supplementary Materials**), which were labelled according to the most representative taxon/taxa (**Supplementary Table S2** in the **Supplementary Materials**). Notably, while *L. crispatus*, *L. jensenii* and *L. gasseri* all belonged to the same CAG, *L. iners* stood apart together with *Ureaplasma*; the two other CAGs were those of taxa more frequently associated to vaginal dysbiosis, comprising *Gardnerella*, *Prevotella*, *Atopobium*, *Dialister* and *Megasphaera* in one and *Streptococcus*, *Porphyromonas*, *Anaerococcus*, and *Finegoldia* in the other.

**Figure 3:**
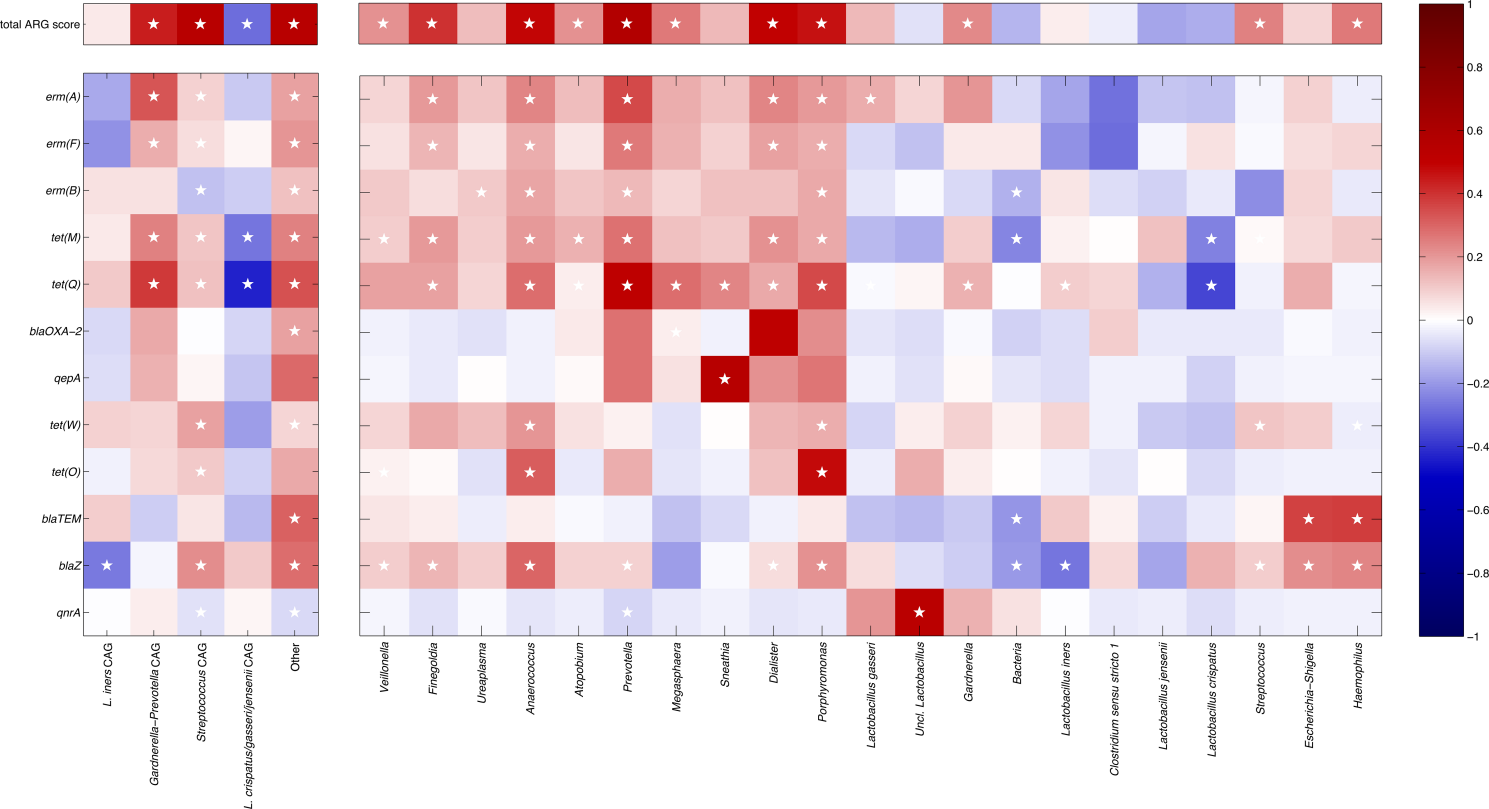
Heatmap showing the correlation coefficients between the relative abundances of the bacterial co-abundance groups (left) and taxa (right) and the total ARGs score (top) and the presence/absence of specific ARGs (bottom). The point-biserial and the Spearman’s correlation coefficients were used for single ARGs and total ARG score, respectively. ARGs and taxa are organized clustering together patterns of similar correlation (as determined by hierarchically clustering according to Pearson’s correlation and average linkage). Color intensity is proportional to the absolute magnitude of correlation. Positive correlations are reported in red, whereas negative ones are in blue. White stars in the heatmap indicate the statistically significant correlations (p ≤ 0.05).

Correlating together the relative abundances of the microbial CAGs and the presence/absence of specific ARGs over all the samples (**Figure 3**) highlighted some interesting features, such as the positive association between several genes related to resistance against macrolides (i.e.: *erm(A)*, *erm(F)*) and tetracycline (i.e.: *tet(M)*, *tet(Q)*) and the *Gardnerella*-*Prevotella* CAG, whereas the *Streptococcus* CAG showed some weak positive correlations, in particular, to *tet(W)* and *blaZ*. On the other hand, the *L. crispatus/jenesenii/gasseri* CAG was negatively associated with all ARGs, particularly to *tet(M)* and *tet(Q)*, whereas the *L. iners* CAG was negatively correlated to *blaZ* resistance. Notably, a positive correlation to quite all the ARGs was observed for the “Other” CAG, which groups together all the low-abundance taxa (i.e.: relative abundance >0.5% in <10% of the samples). At the same time, the *Gardnerella*-*Prevotella*, the *Streptococcus* and the “Other” CAGs were positively correlated to the total ARG score, whereas the *L. crispatus/jenesenii/gasseri* CAG was negatively correlated.

When evaluating single taxa, we found that, among the *Gardnerella*-*Prevotella* CAG, *Prevotella* and *Dialister* showed the higher number of ARGs (*erm(A)*, *erm(B)*, *erm(F)*, *tet(M)*, *tet(Q)*), whereas *Atopobium*, *Gardnerella*, and *Megasphaera* all showed a correlation to *tet(Q)*; all these genera were positively correlated to the total ARG score. At the same time, in the *Streptococcus* CAG, several positive correlations were found for *Finegoldia* (i.e.: *erm(A)*, *erm(F)*, *tet(M)*, *tet(Q)*), *Porphyromonas* and *Anaerococcus* (i.e.: *erm(A)*, *erm(B)*, *erm(F)*, *tet(M)*, *tet(Q)*, *tet(W)*, *tet(O)*, *blaZ*); again, all of them were directly correlated to the total ARG score. Conversely, in the *L. crispatus/jenesenii/gasseri* CAG, *L. crispatus* was negatively associated with all ARGs, particularly to *tet(M)* and *tet(Q)*, while the other lactobacilli in this CAG showed a similar behavior, except with a positive association between *L. gasseri* and *erm(A)*, and between unclassified members of the *Lactobacillus* genus and *qnrA*. Finally, among the bacteria comprised in the “Other” CAG, in particular, *Sneathia* showed a positive correlation to *qepA* and *tet(Q)*, whereas *Escherichia-Shigella* and *Haemophilus* were correlated to *blaTEM* and *blaZ* genes.

## 4 Discussion

Antibiotic resistance represents one of the greatest challenges facing modern healthcare, driven by the widespread use of antibiotics and the consequent dissemination of ARGs. These genes enable bacteria to survive antibiotic treatments, potentially compromising the effectiveness of antibiotic therapies. Investigating ARGs in the vaginal microbiota is therefore of significant relevance for both women’s and public health.

Bacteria in the vaginal flora can develop and transmit drug resistance determinants, particularly against tetracyclines, macrolides and beta-lactams, which are the most commonly used classes of antibiotics. Additionally, resistance to quinolones has recently emerged as a notable and potentially concerning trend.

In this study, we assessed the distribution of selected ARGs conferring resistance to tetracyclines, macrolides, beta-lactams and quinolones within CSTs of 105 Caucasian women in their reproductive age. We investigated the main factors (exposures) potentially linked with the presence of these resistance genes and with an overall composite ARGs score, with a particular focus on individual knowledge and attitudes toward antibiotics, antibiotic consumption and practices, and the characteristics of the vaginal microbiome, particularly its bacterial composition.

In particular, we assessed the presence of 14 ARGs and two related transposons: *erm(A)*, *erm(B)*, *erm(F)*, *tet(M)*, *tet(M)-Tn916*, *tet(O)*, *tet(W)*, *tet(Q)*, *tet(Q)-*rteA, *blaOXA-2*, *blaTEM*, *blaZ*, *blaSHV*, *blaCTX-M*, *qnrA* and *qepA*. The exposures considered included the following demographic, and health-related behavioral data of the participants: age, educational, marital status, BMI, contraceptive use, smoking habits, presence/absence of *Candida* spp., and adherence to a healthy diet such as the Mediterranean diet, individual practice and awareness about antibiotics and antibiotic resistance.

Although a broader panel of ARGs has previously been investigated by other authors using next-generation sequencing (Bostwick et al., 2016), this is the first study to associate the presence of selected ARGs with individual lifestyle variables and antibiotic-related behaviors, in order to highlight their influence on ARG dissemination in the vaginal environment.

Our 16S rRNA sequencing analysis revealed that CSTs I and III were the most prevalent, followed by CST IV, with CSTs II and V being less common. These findings align with previous studies examining the vaginal microbiota in Caucasian reproductive-aged women (Ravel et al., 2011; Tamarelle et al., 2024).

Among the ARGs researched, the most prevalent ones were *erm(F)*, *tet(M)*, *erm(B)*, *erm(A)* and *tet(W)*, while *blaZ*, *tet(Q)* and *blaTEM* were less frequent. Notably, nearly all *tet(M)* and *tet(Q)*-positive samples also harbored their respective mobile elements *Tn916* and *rteA*. These results are in line with previous studies on the gut and cervicovaginal microbiome, which revealed that the most abundant resistance determinants are related to tetracyclines and macrolides (Milanović et al., 2017; Roachford et al., 2021; Fadeyi et al., 2023). Similarly, the co-presence of *tet(M)* and *Tn916* has been previously observed in VMB of pregnant women (Severgnini et al., 2021), suggesting the potential for horizontal gene transfer of tetracycline resistance genes between commensal and pathogenic vaginal bacteria (Chopra and Roberts, 2001).

When exploring the potential associations between the presences of ARGs, both as single gene and as composite ARGs score, and the considered exposures, interesting data emerged. For instance, we found that the use of contraceptives was negatively associated with the presence of *tet(M)*. Research on the impact of oral contraceptives pills (OCP) has predominantly shown an increase in the relative abundance of beneficial *Lactobacillus* species and a reduction in bacterial vaginosis (BV)-associated taxa, thus resulting in a protective effect (Vodstrcil et al., 2013; Brooks et al., 2017). Conversely, copper intrauterine devices (Cu-IUDs) were associated with an increased BV risk (Brown et al., 2023), whereas depot medroxyprogesterone acetate (DMPA) and vaginal rings (VR) showed mixed results (Molatlhegi et al., 2020; Crucitti et al., 2018). All contraceptive users in our cohort reported using OCPs, supporting previous findings of their beneficial effect on vaginal health.

Smoking was positively associated with the presence of *erm(B)* and total ARG score. Bradshaw and colleagues showed that there was a direct and positive correlation between smoking habits and BV, whereby the greatest risk of BV was present in smokers of ≥30 cigarettes per week (Bradshaw et al., 2014). Smoking has been proven to have a negative impact on vaginal microbiota (VMB) associated with an increased risk for multiple and varied bacterial infections (Bagaitkar et al., 2008; Brotman et al., 2014). To our best knowledge, our work is the first to report an independent association between smoking habit and antibiotic resistance in the vaginal environment, although an increased AR for smokers has been previously reported for urinary tract infections treatments (Lorenzo-Gómez et al., 2020).

Similar data were observed with BMI. A higher BMI was positively associated with *erm(A)*, *tet(O)* and total ARG score. Although the cohort had similar BMI values in the normal range, this association reflects previous findings linking higher BMI with increased ARG prevalence in the first trimester of pregnancy (Severgnini et al., 2021). Additionally, a higher MEDILITE score was negatively associated with *tet(O)*. Our findings suggest that maintaining an appropriate body weight and adhering to a balanced diet are beneficial factors in preserving a healthy vaginal microbiome.​ Thoma and colleagues’ study on a cohort of 1735 nonpregnant women, found that those who consumed healthier diets were less likely to develop BV (Thoma et al., 2011). Moreover, a recent study that examined the relationship between dietary intake and VMB depicted how higher consumption of low-fat dairy, fruit, vitamin D, yogurt, and fiber were associated with a *L. crispatus*-dominated CST (Rosen et al., 2022).

Antibiotic-related behaviors emerged as influential factors: indeed, inappropriate use and low compliance were associated with increased ARG prevalence, whereas past compliance or higher awareness of antibiotic resistance were negatively associated with selected ARGs. A direct correlation between an increase in antibiotic use, mainly driven by inappropriate use and an increase in ARGs has been already reported (Cantón et al., 2013; Kunhikannan et al., 2021). These findings reinforce the importance of appropriate antibiotic use in limiting ARG dissemination. Besides, the presence of *Candida* spp. was significantly positively associated with the total ARG score, supporting existing evidence that antibiotics can promote *Candida* spp. overgrowth by disrupting the bacterial microbiota (Spinillo et al., 1999; Armstrong et al., 2024).

Alpha-diversity increased in tandem with the number of ARGs per sample, and higher ARG burden was linked to the enrichment of BV-associated bacterial taxa. In particular, *Anaerococcus*, *Prevotella* and *Dialister* were positively associated with resistance genes against macrolides and tetracyclines. It is worth mentioning that bacteria from different sites appear to be exchanging genetic material (Salyers et al., 2004). Also, some of the bacteria associated with the presence of ARGs in this study are commensal bacteria present in the gut microbiota and it is well-established that the human gut microbiota is an important reservoir for AR (Francino, 2016; Salyers et al., 2004). Thus, we can speculate that a bacterial translocation from the gastrointestinal-tract to the vagina through the gut-vagina axis has occurred (Amabebe and Anumba, 2020; Takada et al., 2023).

On the other hand, the genus *Lactobacillus* was negatively associated with all ARGs, with a few exceptions: positive association between *L. gasseri* and *erm(A)*, *L. iners* and *tet(Q)* and, between unclassified members of the *Lactobacillus* genus and *qnrA*. This suggests that even commensal and health-promoting bacteria may acquire and harbor ARGs (Fadeyi et al., 2023).

This aspect has been highlighted by several studies suggesting that lactobacilli adapt to their environment by acquiring resistance genes from other bacteria through horizontal gene transfer. This phenomenon is exacerbated by several factors, such as the increasingly selective pressure exerted by antimicrobial treatments on lactobacilli colonizing the human gastrointestinal and vaginal tracts, as well as the widespread use of antibiotics in the food chain, where lactobacilli are often intentionally added as starter cultures (Campedelli et al., 2018; Colautti et al., 2022; Huang et al., 2024).

In this context, numerous studies have investigated how lactobacilli may act as reservoirs of antibiotic resistance genes that could potentially be transferred to pathogenic species (Campedelli et al., 2018; Colautti et al., 2022; Fadeyi et al., 2023). The most commonly studied resistances are those to tetracyclines and macrolides, associated with *tet* and *erm* genes, respectively (Colautti et al., 2022). Further studies are needed to better understand the role of *Lactobacillus* species as ARG reservoirs in relation to different CSTs (i.e., *L. crispatus*, *L. gasseri*, *L. iners*, and *L. jensenii*).

Other interesting results emerged when correlating together the relative abundances of microbial CAGs and the presence of ARGs. In particular, the *L. crispatus/jenesenii/gasseri* CAG was negatively correlated to the total ARG score, thus representing a potential ‘protective’ vaginal *Lactobacillus* community (Djusse et al., 2025). On the other hand, *L. iners* CAG exhibited neither positive nor negative correlation, strengthening the idea that the role of this *Lactobacillus* species in vaginal health remains unclear (Petrova et al., 2017). Indeed, *L. iners* has been considered a ‘transitional’ poorly protective species, typically associated with dysbiotic conditions. At the same time, it can be detected in normal conditions in a large subset of women, being its presence associated with young age and unprotected sexual practices (Novak et al., 2022).

Overall, our research delves into the complexity of the dynamics between the distribution of a panel of ARGs in the VMB of women in their reproductive age, with a particular emphasis on resistance to tetracyclines, macrolides, beta-lactams and quinolones. The high prevalence of ARGs such as *erm(F)*, *tet(M)*, *erm(B)* along with mobile genetic elements (transposons), underscores the potential for horizontal gene transfer within the vaginal ecosystem. Importantly, we identified several health-related behavioral, clinical, and demographic factors like contraceptive use, smoking, BMI, diet, presence of *Candida* spp. and antibiotic behavior that were significantly associated with the presence or absence of specific ARGs and to the total number of ARGs. These correlations reinforce the impact of lifestyle, health and antibiotic consumption on dynamics of antibiotic resistance. Notably, our findings suggest a protective role for oral contraceptives and healthy dietary patterns, while highlighting smoking and inappropriate antibiotic use as risk factors.

Taxonomic analysis further revealed a clear relationship between the presence of ARGs and an increase in bacterial diversity, particularly with the enrichment of BV-associated bacteria, while *Lactobacillus* spp. was negatively associated with ARG abundance. Interestingly, a few ARGs were also associated with commensal bacteria, suggesting that ARG dissemination may occur even in the absence of obvious risk factors.

Together, these findings emphasize the need for integrated public health strategies that combine antibiotic stewardship with broader lifestyle and behavioral interventions. Individualized therapeutic approaches, based on an individual’s resistome, may also represent a promising avenue for future treatment strategies.

We are fully aware of some limitations of this study. At first, even though we accounted for key factors potentially related to the distribution of ARGs in the VMB such as antibiotic behavior, contraceptive methods, smoking habits and diet, residual confounding cannot be excluded, especially regarding variables such as sexual behavior and microbiota exchanges with partners. Also, a shotgun metagenomic sequencing approach could provide a better comprehension of the resistance profiles actually present in some specific bacterial species. Additionally, the cross-sectional design limits causal inference, as both exposures and outcomes are measured simultaneously, thus it is difficult to establish a clear temporal sequence. Lastly, there is a sampling limitation, as the sample is not representative of the general Italian population. It consists of young women of reproductive age who voluntarily participated in the study and have a high level of education. This intrinsic selection bias substantially limits the generalizability of the findings, which should, therefore, be interpreted with caution. In particular, the results cannot be extrapolated to women of different age groups or educational backgrounds.

These findings lay the groundwork for longitudinal and functional studies to: (i) clarify causal relationships, (ii) explore ARG transmission via the gut-vagina axis, (iii) enable strain-level characterization of ARG-harboring bacteria through targeted bacterial isolation, (iv) analyze the functional activity/expression of detected ARGs, (v) assess a wider panel of genes, including those conferring resistance to 5-nitroimidazole, an antimicrobial commonly used in the vaginal setting (Williams et al., 2025).

## Data Availability

Raw sequencing data for this project are available in NCBI Short-Read Archive (SRA) under accession number PRJNA1188525 (https://www.ncbi.nlm.nih.gov/bioproject/PRJNA1188525).
