# Supplementary material for: Linking antibiotic resistance genes in the vaginal microbiota to health-related behaviors and antibiotic awareness in reproductive-age women: a cross-sectional study
Source: Front Cell Infect Microbiol. 2025 Sep 18;15:1640992. doi: 10.3389/fcimb.2025.1640992 (PMC12488730; doi:10.3389/fcimb.2025.1640992)
Supplement: Supplementary file 1 [file DataSheet1.pdf]

SUPPLEMENTARY MATERIAL

Linking Antibiotic Resistance Genes (ARGs) in the vaginal microbiota to Health-related Behaviors and Antibiotic Awareness in Reproductive-Age Women: A Cross-Sectional Study

Paola Castellano[1^], Camilla Ceccarani[2,3^], Marielle Ezekielle Djusse[4,5^], Michela Mazzetti[1], Sara Morselli[4], Tania Camboni[2], Silvia Conti[2,6], Federica Prinelli[2], Marco Severgnini[2,3], Claudio Foschi[4,7*], Margherita Dall'Asta[8], Clarissa Consolandi[2,3£], Antonella Marangoni[4£]

[1]Department of Medical and Surgical Sciences (DIMEC), Alma Mater Studiorum - University of Bologna, Bologna, Italy.

[2]Institute of Biomedical Technologies, National Research Council, Segrate, Italy.

[3]National Biodiversity Future Center S.c.a.r.l., Palermo, Italy.

[4]Section of Microbiology, Department of Medical and Surgical Sciences (DIMEC), Alma Mater Studiorum - University of Bologna, Bologna, Italy.

[5]International PhD College, Collegio Superiore of Alma Mater Studiorum, University of Bologna, Bologna, Italy.

[6]Department of Medical Sciences, University of Ferrara, Ferrara, Italy.

[7]Microbiology Unit, IRCCS Azienda Ospedaliero-Universitaria di Bologna, Bologna, Italy.

[8] Department of Animal Science, Food and Nutrition (DIANA), Università Cattolica Del Sacro Cuore, Piacenza, Italy.

^These three authors contributed equally to this work and share first authorship. Author order was determined on the basis of alphabetical order.

£These two authors contributed equally to this work and share last authorship. Author order was determined on the basis of alphabetical order.

Figure S1. Principal coordinate analysis (PCoA) plots based on the unweighted UniFrac distance among samples.

Points represent single samples, colored, in each plot, according to presence or absence of the specific ARG; centroids represent the average coordinate of the samples within the same category and ellipses are the SEM-based confidence intervals; for each plot, the first and second coordinate are represented.

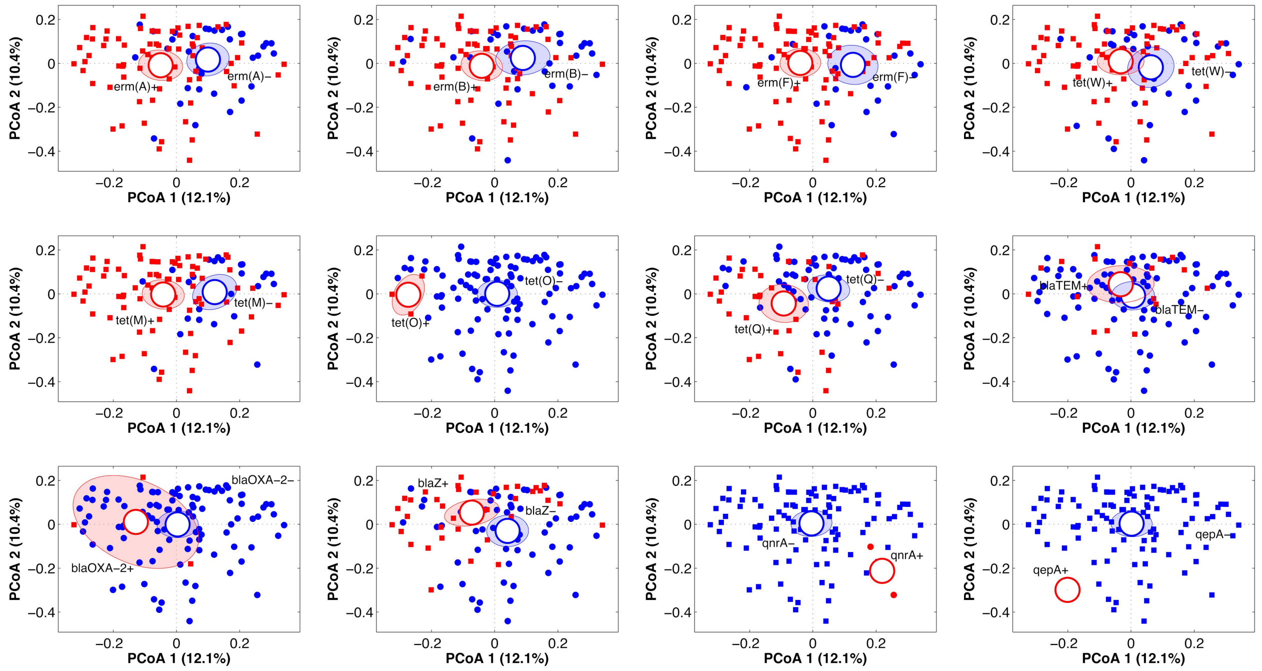

Figure S2. Boxplots of the significantly different taxa over the categorized ARG score. Box and whiskers plot represent the median + IQ range of the data distribution; median is indicated as a red line, whereas the distribution mean is indicated as a blue line. For each taxa, individual abundances are highlighted as gray dots. Statistically different taxa are highlighted by black "*" above the boxplots. "Rel.ab(%)": relative abundance. For visualization reasons, abundances are represented in $\log_{10}$ scale.

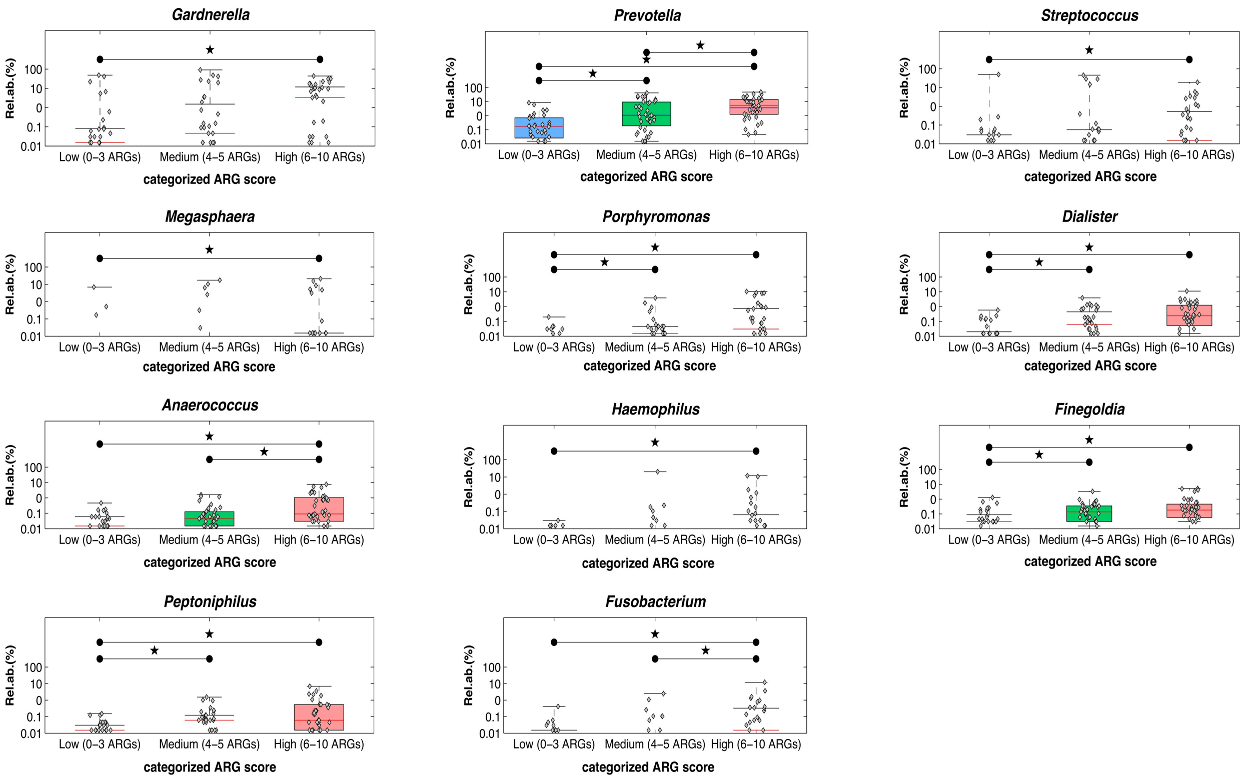

Figure S3. Taxa co-abundance groups (CAGs) analysis plots. (A) Heatmap showing the clusters of co-abundant taxa; heatmap color is proportional to the Spearman's rank correlation among taxa and is clustered according to the Pearson distance and average linkage. Only taxa with an abundance >0.5% in >10% of samples (n=11) over the entire dataset (n=105) were used. The dendrogram on the right highlights the four CAGs obtained. (B) Barplots of the CAGs abundance over the entire dataset. CAGs are named according to the most representative taxon/taxa. The "Other" group comprises all the taxa not used for the CAG heatmap.

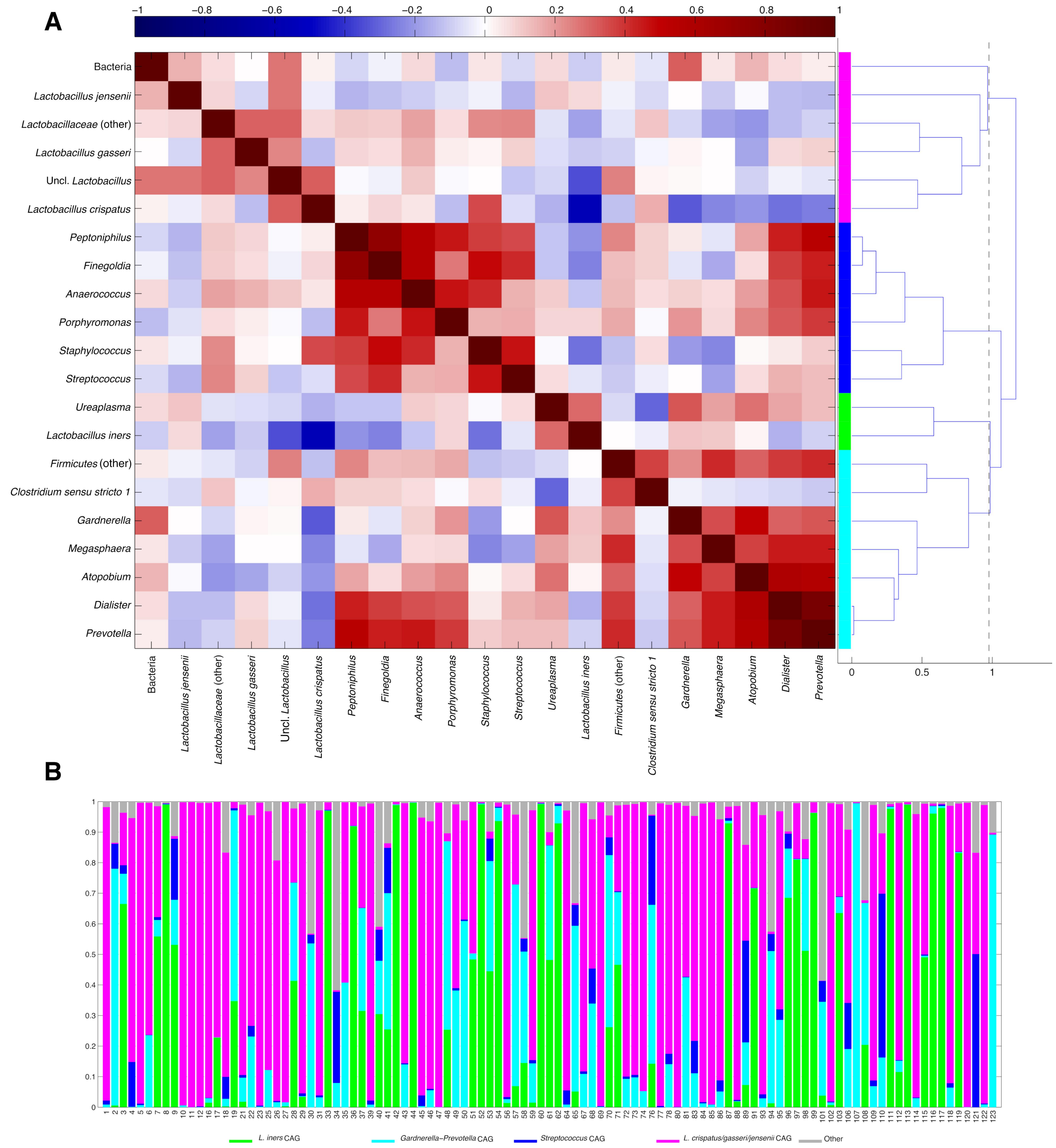

Table S1. List of primer sequences and PCR conditions for the detection of ARGs with modifications. The amplicons were observed on 1%, 1.5% and 2% gel electrophoresis based on their band size.

**Gene**	**Primer sequences**	**PCR conditions**	**Amplicon size**	**References**
erm(A)	F 5′-CCCGAAAAATACGCAAAATTTCAT-3′<br>R 5′-CCCTGTTTACCCATTTATAAACG-3′	95°C for 5 min; 30 cycles of 95°C for 1 min, 59°C for 1 min and 72°C for 1 min 30 sec; 72°C for 10 min	590 bp	[1]
erm(B)	F 5’-GAAAAGGTACTCAACCAAATA-3’<br>R 5’-AGTAACGGTACTTAAATTGTTTAC-3’	95°C for 10 min; 35 cycles of 94°C for 1 min, 54°C for 1 min and 72°C for 1 min; 72°C for 7 min	639 bp	[2]
erm(F)	F 5’-CGGGTCAGCACTTTACTATTG-3’<br>R 5’-GGACCTACCTCATAGACAAG-3’	95°C for 10 min; 35 cycles of 94°C for 1 min, 48°C for 1 min and 72°C for 1 min; 72°C for 7 min	466 bp	[3, 8]
tet(M)	F 5’-ACCCGTATACTATTTCATGCACT-3’<br>R 5’-CCTTCCATAACCGCATTTTG-3’	95°C for 10 min; 35 cycles of 94°C for 1 min, 48°C for 1 min and 72°C for 2 min; 72°C for 7 min	1115 bp	[2]
tet(M)-Tn916	F 5’-TACTACCGGTGAACCTGTTTGCCA-3’<br>F 3’-TTTAGCCAGCGGTATCAACGAAGC-5’	95°C for 10 min; 35 cycles of 94°C for 1 min, 55°C for 1 min and 72°C for 1 min; 72°C for 7 min	472 bp	[4]
tet (O)	F 5’-ACGGARAGTTTATTGTATACC-3’<br>R 5’-TGGCGTATCTATAATGTTGAC-3’	94°C for 5 min;30 cycles of 94°C for 30 sec, 51°C for 30 sec and 72°C and 30 sec; 72°C for 7 min	171 bp	[5]
tet(w)	F 5’-GAGAGCCTGCTATATGCCAGC-3’<br>R 5’-GGGCGTATCCACAATGTTAAC-3’	95°C for 10 min; 35 cycles of 94°C for 1 min, 62°C for 1 min and 72°C per 1 min; 72°C for 7 min	168 bp	[2]
tet(Q)	F 5’-GATACICCIGGICAYRTIGAYTT-3’<br>R 5’-GCCCARWAIGGRTTIGGIGGIACYTC-3’	95°C for 10 min; 35 cycles of 94°C for 1 min, 57°C for 1 min and 72°C for 1 min; 72°C for 7 min	904 bp	[2]
tet(Q)-rteA	5’-TGTGGACATTCTCGAACCGATGCT-3’<br>5’-CTATCTCCTGCCATTCATAGAGGC-3’	95°C for 10 min; 35 cycles of 94°C for 1 min, 60°C for 1 min and 72°C and 1 min; 72°C for 7 min	435 bp	[4]
blaOXA-2	F 5’-TTCAAGCCAAAGGCACGATAG-3’<br>R 5’-TCCGAGTTGACTGCCGGGTTG-3’	96°C for 5 min; 35 cycles of 96°C for 1 min, 65°C for 1 min and 72°C for 2 min; 72°C for 10 min	702 bp	[6]
blaTEM	F 5’-TTTCGTGTCGCCCTTATTCC- 3’<br>R 5’-CCGGCTCCAGATTTATCAGC-3’	94°C for 5 min; 30 cycles of 94°C for 30 sec, 60°C for 30 sec and 72°C for 1 min; 72°C for 5 min	692 bp	[7]
blaZ	F 5′-ACTTCAACACCTGCTGCTTTC-3′<br>R 5′-TGACCACTTTTATCAGCAA-3′	95°C for 5 min; 30 cycles of 95°C for 1 min, 59°C for 1 min and 72°C for 1 min 30 sec; 72°C for 10 min	173 bp	[1]
blaSHV	F 5′-TCGCCTGTGTATTATCTCCC-3′<br>R 5′-CGCAGATAAATCACCACAATG-3′	94 °C for 5 min; 30 cycles of 94 °C for 30 sec, 50 °C for 30 sec, and 72 °C for 90 sec; 72 °C for 10 min	768 bp	[1]
blaCTX-M	F 5′-TTTGCGATGTGCAGTACCAGTAA-3′<br>R 5′-CGATATCGTTGGTGGTGCCATA-3′	94 °C for 5 min; 30 cycles of 94 °C for 30 sec, 56 °C for 1 min and 72 °C for 60 sec; 72 °C for 10 min	543 bp	[1]
qnrA	F 5′-ATTTCTCACGCCAGGATTTG-3′<br>R 5′-GATCGGCAAAGGTTAGGTCA-3′	94 °C for 2 min; 35 cycles of 94 °C for 30 sec, 53 °C for 30 sec and 72 °C for 60 sec; 72 °C for 10 min	516 bp	[1]
qepA	F 5′-CTTCTCTGGATCCTGGACAT-3′	94 °C for 2 min; 35 cycles of 94 °C for 30 sec, 54 °C	720 bp	[1]

	R 5′-TGAAGATGTAGACGCCGAAC-3′	for 30 sec and 72 °C for 60 sec; 72 °C for 10 min		

References

1. Fadeyi TE, Oyedemi OT, Awe OO, Ayeni F. Antibiotic use in infants within the first year of life is associated with the appearance of antibiotic-resistant genes in their feces. PeerJ. 2023;11:e15015. doi: 10.7717/peerj.15015.

2. Milanović V, Osimani A, Aquilanti L, Tavoletti S, Garofalo C, Polverigiani S, Litta-Mulondo A, Cocolin L, Ferrocino I, Di Cagno R, Turroni S, Lazzi C, Pellegrini N, Clementi F. Occurrence of antibiotic resistance genes in the fecal DNA of healthy omnivores, ovo-lacto vegetarians and vegans. Mol Nutr Food Res. 2017;61(9). doi: 10.1002/mnfr.201601098.

3. Sirichoat A, Flórez AB, Vázquez L, Buppasiri P, Panya M, Lulitanond V, Mayo B. Antibiotic Susceptibility Profiles of Lactic Acid Bacteria from the Human Vagina and Genetic Basis of Acquired Resistances. Int J Mol Sci. 2020;21(7):2594. doi: 10.3390/ijms21072594.

4. Jeters RT, Rivera AJ, Boucek LM, Stumpf RM, Leigh SR, Salyers AA. Antibiotic resistance genes in the vaginal microbiota of primates not normally exposed to antibiotics. Microb Drug Resist. 2009;15(4):309-15. doi: 10.1089/mdr.2009.0052.

5. Gueimonde M, Salminen S, Isolauri E. Presence of specific antibiotic (tet) resistance genes in infant faecal microbiota. FEMS Immunol Med Microbiol. 2006;48(1):21-5. doi: 10.1111/j.1574-695X.2006.00112.x.

6. Steward CD, Rasheed JK, Hubert SK, Biddle JW, Raney PM, Anderson GJ, Williams PP, Brittain KL, Oliver A, McGowan JE Jr, Tenover FC. Characterization of clinical isolates of *Klebsiella pneumoniae* from 19 laboratories using the National Committee for Clinical Laboratory Standards extended-spectrum beta-lactamase detection methods. J Clin Microbiol. 2001;39(8):2864-72. doi: 10.1128/JCM.39.8.2864-2872.2001.

7.Bailey JK, Pinyon JL, Anantham S, Hall RM. Distribution of the blaTEM gene and blaTEM-containing transposons in commensal *Escherichia coli*. J Antimicrob Chemother. 2011;66(4):745-51. doi: 10.1093/jac/dkq529.

8. Roberts MC, Chung WO, Roe D, Xia M, Marquez C, Borthagaray G, Whittington WL, Holmes KK. Erythromycin-resistant *Neisseria gonorrhoeae* and oral commensal *Neisseria* spp. carry known rRNA methylase genes. Antimicrob Agents Chemother. 1999;43(6):1367-72. doi: 10.1128/AAC.43.6.1367.

Table S2. List of taxa in each co-abundant group (CAG). CAGs are named according to the most representative taxon/taxa. See Figure S3 for more details.

**CAG**	**Taxa**
L. iners CAG	*Lactobacillus iners*<br>*Ureaplasma*
Gardnerella-Prevotella CAG	*Gardnerella*<br>*Prevotella*<br>*Atopobium*<br>*Megasphaera*<br>*Dialister*<br>*Clostridium sensu stricto 1*<br>*Other Firmicutes*
L. crispatus/gasseri/jensenii CAG	*Lactobacillus crispatus*<br>*Lactobacillus gasseri*<br>*Lactobacillus jensenii*<br>Unclassified *Lactobacillus*<br>Other *Bacteria*<br>Other *Lactobacillaceae*
Streptococcus CAG	*Streptococcus*<br>*Porphyromonas*<br>*Anaerococcus*<br>*Finegoldia*<br>*Peptoniphilus*<br>*Staphylococcus*
